# Current Status of *Pulsatilla patens* in Latvia—Population Size, Demographic and Seed Viability Indicators, Soil Parameters and Their Relationships

**DOI:** 10.3390/plants14030375

**Published:** 2025-01-26

**Authors:** Dace Kļaviņa, Anita Osvalde, Guntis Tabors, Gunta Jakobsone

**Affiliations:** 1 National Botanic Garden of Latvia, 1 Miera Street, LV-2169 Salaspils, Latvia; dace.klavina@nbd.gov.lv (D.K.); gunta.jakobsone@gmail.com (G.J.); 2 Institute of Biology, Faculty of Medicine and Life Sciences, University of Latvia, 4 O. Vaciesa Street, LV-1004 Riga, Latvia; 3 Faculty of Medicine and Life Sciences, University of Latvia, 1 Jelgavas Street, LV-1004 Riga, Latvia; guntis.tabors@lu.lv

**Keywords:** Eastern pasque flower, population monitoring, demographic assessment of *P. patens*, seed viability, soil types, nutrient content in soil

## Abstract

*Pulsatilla patens* (L.) Mill. (Eastern pasque flower) is classified as a highly endangered and declining species in Europe. The present research assessed the current status of *P. patens* in Latvia by collecting data on its distribution in historical places, Natura 2000 territories, and other areas, largely covering the entire country. We aimed to analyze the relationships between *P. patens* populations size, demographic indicators, and soil parameters, in order to gain knowledge on the impact of local ecological factors and optimal growth conditions, which are important for conservation and potential reintroduction. Although *P. patens* was not detected in more than a third of the surveyed 624 locations, more than 18 thousand individuals were recorded. Our results indicate that optimal growth conditions for *P. patens* occurred near highways, forest roads, and paths, that is, in places with reduced competition from other species and improved lighting conditions. The seed viability ranging from 22% to 62% can be considered potentially sufficient for the continuation of the species if enough flowering plants and moss-free spaces for germination are maintained. Although *P. patens* tolerates a broad soil pH range, in Latvia this species mainly grows in acidic sandy soils with an average pH_KCl_ of 4.07. The soil parameters that most strongly positively correlated with *P. patens* regional population size and performance included higher soil pH level and plant available nutrient content, particularly P, K, Ca, Mg, Mn and B. Increased soil P and Mn levels significantly enhanced flowering, while high organic matter content could be associated with reduced population sizes. Despite its still large current population, long-term risks remain without active management. Conservation measures, such as creating open soil areas, where vegetation is removed and shading is reduced, are necessary to mitigate population decline.

## 1. Introduction

Estimates suggest that 45% of the world’s flowering plant species are potentially endangered and may be at risk of extinction [1]. Such predictions are based on a range of factors, primarily geographic distribution, life-history traits, human impact, and climate change. Globally, ambitious goals and objectives have been set to stop plant species extinction, achieve stabilization and create solutions for recovery [2]. However, these goals remain unmet. Area-based conservation, site-focused conservation and restoration efforts, and active management to enable species recovery were proposed as the main targets in the post-2020 Global Biodiversity Framework [3,4]. Achieving these targets requires thorough knowledge of the status of the specific species locally, in the country, the distribution dynamics, and the impact of various environmental factors on their populations.

*Pulsatilla patens* (Eastern pasque flowers) is protected as a rapidly declining species in all European countries where it occurs. It is protected by Annex I of the Berne Convention, listed in Annexes II and IV of the European Union Habitats Directive, and as a vulnerable species in the new Red Data Book (RDB) of Latvia and protected by law. *P. patens* is a light-demanding, long-lived, disturbance-dependent perennial species, which reproduces mainly by seeds and lacks a persistent seed bank [5,6,7]. Low competitiveness with other understory plant species, lack of natural disturbances in forest ecosystems [8,9], and destruction of flowers and fruit shoots by animals [10] are among the reasons why *P. patens* populations are endangered. It is possible that the decline of the species is largely due to changes in land use [11]. Cessation of livestock grazing, modern forestry practices and highly effective fire prevention have led to understory vegetation compaction in forests. For successful reproduction of *P. patens*, maintenance of habitat heterogeneity and prevention of interlocking of understory vegetation is important [9]. Litter accumulation has been found to be a major factor affecting seedling development and population survival [9]. Deforestation, which has increased significantly in recent years, is a major factor destroying the habitats of *Pulsatilla* species. Heavy machinery destroys the rhizomes, while modified soils promote the invasion of ruderal species or light-demanding cereals, forming a dense vegetation cover, under which it is impossible for *Pulsatilla* seedlings to survive [7]. Sand mining and burrowing by wild boars are also additional factors limiting the population [12].

Determining favorable environmental conditions for endangered plant species is a challenging research task due to its complexity. Among many others, soil conditions are an important factor in the growth and persistence of the species. It was found that the traits of *P. patens* populations could be determined by the degree of soil acidity and the fertility of the upper soil layer [13]. However, available data on suitable soil parameters are often general or contradictory. For instance, based on research in NE Poland, *P. patens* thrives in soils with a low soil reaction, with a pH between 4 and 5 [13], while in Southern Poland it grows successfully in soil with a pH level of 7.4–7.6 [14]. In Estonia, a higher soil pH, ranging from 5.1 to 8.4, was reported for *P. patens* populations [7], in Yellowstone National Park in the USA from 5.0 to 7.1 [15], and in Canada from 6.1 to 7.0 [16]. In general, this species favors nutrient-poor soils but has a rather wide range of tolerance to soil nutrient conditions [7,16,17]. However, several studies also indicated the relationship between the most nutrient-rich growth environment and *P. patens* individuals with the highest morphological-developmental characteristics [13,18,19]. Therefore, more detailed studies on soil properties and nutrient status are needed to not only understand the ecology of *P. patens*, but also to find out the optimal growth conditions, which are especially important for conservation and possible reintroduction needs. Currently, there are limited data on soil characteristics for *P. patens* populations in Latvia [20]. Given this lack of knowledge, it is critical to ascertain all ecological factors, including soil that may affect the population of this endangered species.

In Latvia, *P. patens* occurs mainly in light boreal forests dominated by *Pinus sylvestris* with *Betula pendula*, *Frangula alnus*, *Quecus robur* and *Sorbus aucuparia* in the understory. In wooded areas where *P. patens* grows, the accompanying herbaceous and small shrub layer consists mainly of *Vaccinium myrtillus*, *Calluna vulgaris*, *Vaccinium vitis-idea*, *Festuca ovina*, and the moss layer is dominated by *Hylocomium splendens* and *Pleurosium schreberi* [20].

In order to better understand the generative (biological) potential of *P. patens* populations in Latvia, the demographic parameters characterizing the population structure are also an important factor. As *P. patens* reproduces mainly generatively [9,21], the future of each population largely depends on flowering individuals. Many studies have shown that the available area for seed germination, characterized by a low thickness of moss and litter layer and bare ground cover, is a factor that stimulates the emergence of juvenile individuals, thereby influencing the structure of the population [9,22,23].

This study aims to clarify the current status of *P. patens* in Latvia by, collecting data on its distribution in historical places, Natura 2000 territories, and other areas, largely covering the entire country. The relationships between *P. patens* population sizes and soil parameters were estimated, to gain knowledge on the impact of local ecological factors and optimal growth conditions, which are particularly important for conservation of the species and potential reintroduction. Demographic parameters were also analyzed to identify factors contributing to population differences. It was hypothesized that, although *P. patens* has been found to have a rather broad tolerance to soil nutrient status, specific soil physicochemical properties could explain variation in population performance.

## 2. Materials and Methods

**Figure 1:**
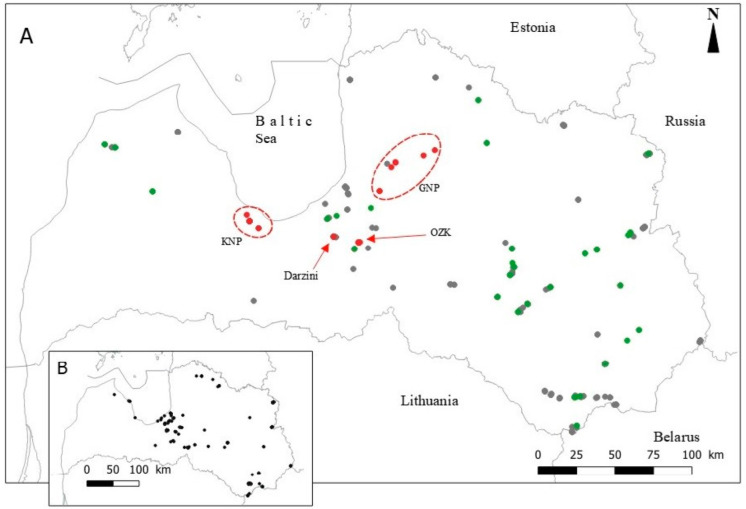
Surveyed sites for the collection of demographic data of *Pulsatilla patens* in Latvia ((**A**): red points—permanent plots; green points—plots surveyed once; grey points—other sites where individuals were recorded, (**B**)—sites where the species is extinct). OZK—Ogres Zilie Kalni Nature Park; GNP—Gauja National Park; KNP—Kemeri National Park. Map created by Z. Striķe.

To assess the state of *P. patens* in Latvia, we conducted a study of populations and updated data. We surveyed the species both in protected territories and outside them. The current and historical sites (data on *P. patens* were collected 20–30 years ago) to be surveyed were coordinated with the Latvian Nature Protection Board, which included reviewing the Latvian State Forests database [24], the Nature Data Management System OZOLS database [25], and questionnaire data. Based on this data and by identifying new locations where the species occurs, a set of surveyed sites was created. The sites were surveyed in the field and fixed with GPS coordinates (Figure 1).

### 2.1. Establishment of Sample Plots

Research on *P. patens* was started in 2010, near the railway station Darzini, Riga by selecting a 40 × 40 m sample plot divided into 5 m wide and 40 m long squares parallel to the highway and railway. In 2015, two demographic survey plots (10 × 10 m) with marked individuals were established in Darzini (Darzini, D): one in the previously selected large plot with the highest concentration of *P. patens* and the other permanent plot on the slope facing the railway (Table 1). Annual census data were collected from these plots until 2021.

Based on an inventory of available data of *P. patens* in Gauja National Park (GNP) and within the framework of a research project in 2017, 18 demographic accounting permanent plots (10 × 10 m) with *P. patens* were established: seven plots in the GNP and 11 in Ogres Zilie kalni Nature Park (OZK). In 2019–2020, subsequent research project established seven sample plots in Kemeri National Park (KNP) (Table 1).

In all 27 permanent plots of these four territories (Figure 1—red points) *P. patens* individuals were marked with plastic label. The plots were marked with four large wooden stakes at the corners connected by string. After surveying, the string was removed and the large stakes were replaced with short stakes to ensure accurate retrieval of the plot during subsequent surveys.

In addition, 33 Natura 2000 territories and 39 territories outside of them were surveyed in 2020–2021. In the Natura 2000 territories, 15 sample plots (10 × 10 m) were selected for demographic assessment. Outside of Natura 2000 sites, 18 sample plots were selected (Figure 1—green points, Table S1).

In 2022, additional surveys of *P. patens* (without establishment of plots) were conducted near Daugavpils at three locations, as well as a site near Lube in northwest Latvia, a region where the species is scarce (Table S1).

### 2.2. Collection of Demographic Data

Each year (over 3 to 10 years), demographic censuses were carried out twice in 27 permanent plots (Figure 1—red points). In spring, the number of generative individuals and the number of flowers were counted; in summer, the number of leaves per individual, including the vegetative ones, was counted. If the distance between leaf rosettes was less than 10 cm, they were counted as one individual; if the distance exceeded 10 cm, they were counted as separate individuals, as indicated by Kalliovirta et al. [9]. Obtained data were used to compare the dynamics of the species in the permanent areas.

In the 33 sample plots surveyed once (Figure 1—green points), the inventory was carried out only once without marking the individual plants. In 2020, the creation of these plots was adapted to record all *P. patens* individuals, including vegetative ones, and to monitor and possibly collect seeds (end of May to the first decade of June, according to 2019 observations). In 2021, five additional plots in Latgale were established during flowering of *P. patens*.

To assess the possible correlation between *P. patens* demographic indicators and soil parameters, only data from 2020–2021, obtained simultaneously with soil sampling, were used.

### 2.3. Determination of Soil Morphology and Chemical Analyses of Soil Samples

For morphological study of the soil in the sample plot, soil drilling was carried out until the parent material (C horizon) was reached, which is the raw material of soil formation, and which determines subsequent soil formation processes. Soil types and subtypes were determined based on the obtained soil horizon parameters using the soil genetic classification system [26]. Soil samples for the analysis of chemical composition were collected using a soil auger from the upper topsoil horizon, known as the organic horizon (O horizon), as done in other previously described studies [27,28]. Each sample consisted of five thoroughly mixed subsamples taken from the plot’s four corners and center. Soil was air-dried and sieved through a 2 mm sieve. Plant-available nutrient concentrations (N, P, K, Ca, Mg, S, Fe, Mn, Zn, Cu, Mo, and B) were determined using a 1 M HCl extraction (soil–extractant volume ratio 1:5) as described by previous studies [29]. Levels of K, Na, Ca, Mg, Fe, Cu, Zn, and Mn were analyzed by microwave plasma atomic emission spectrometer (MP-AES, Agilent 4200; Agilent Technologies, Santa Clara, CA, USA), while N, P, Mo, and B were measured by colorimetry, and S by turbidimetry (spectrophotometer JENWAY 6300; Barloworld Scientific Ltd., Dunmow, Essex, UK). All the results of nutrients were expressed as mg L^−1^. Soil pH was measured in a 1 M KCl soil-extractant mixture (1:2.5) using a pH-meter (Sartorius Basic Meter PB-20; Sartorius AG, Gottingen, Germany). Soil electrical conductivity (EC, mS cm^−1^) was measured in soil-distilled water mixture (1:5) with the conductivity meter (Hanna Instruments 215 EC, Guangzhou, China). Soil organic matter content (%) was determined using the Tjurin method with potassium dichromate oxidation.

### 2.4. Seed Viability Analysis

Viability of seeds, collected in the expeditions, was analyzed only for those plots where the seeds were fully mature. Thus, seed viability data were not obtained for all surveyed sites. Aggregated data for all sample plots and study years were used to assess seed viability of each study area. All seeds were first counted, sorted after pappus removal and visually developed seeds were taken for viability tests. Seed viability was determined using 1% 2,3,5-triphenyltetrazolium chloride test, a method developed for orchids [30] and approbated for *P. patens* seeds (G. Jakobsone, NBG, Department of Plant Ecophysiology). Viable seeds stained red, while non-viable ones remained white or light pink. The results were expressed as the percentage of viable seeds from the total number of seeds.

### 2.5. Statistical Analysis

Data on soil chemical composition and demographic assessment of *P. patens* were analyzed using descriptive statistics. The coefficient of variance (CV) was calculated to characterize the heterogeneity of soil indices. Student’s t-test and ‘Two-Sample Assuming Unequal Variances’ (*p* < 0.05) with Bonferroni correction was used to test the significance of differences in soil characteristics and demographic parameters between *P. patens* sites. Pearson’s correlation coefficients were calculated to assess the correlation between soil parameters and the total number of *P. patens* individuals, as well as the number of flowering individuals per 100 m^2^ plot, using data from all study sites. In addition to correlation analysis, linear regression analysis was performed to examine the relationship between the number of individuals and soil parameters, as well as between the number of leaves in the previous year and flowering in the subsequent year. Principal component analysis (PCA) was performed on the mineral nutrition data, grouping the study sites by location of permanent plots (OZK, GNP, KNP, Darzini) and two population groups of different *P. patens* density. Seven sites with low density (i.e., less than 10 individuals per 100 m^2^) and seven sites with high density (i.e., more than 100 individuals per 100 m^2^) were selected from the other 33 study sites (excluding OZK, GNP, KNP, and Darzini).

## 3. Results

### 3.1. P. patens Population Assessment in Latvia

In total, 342 locations were surveyed across 33 Natura 2000 territories, where *P. patens* had been previously detected. However, in 10 of these Natura 2000 territories and in a total of 115 sites *P. patens* was not found. In the remaining areas, 6629 individuals were recorded with 4702 counted by the research team and 1927 by other experts at five incompletely surveyed areas. The data are shown in Table S1. In the Ogres Zilie kalni NP (OZK), an average of 822 individuals of *P. patens* were counted in 11 sample plots over five years, with a total of 854 counted, including roadside plants outside the sample plots. OZK is probably the most abundant population of *P. patens* of the Natura 2000 territories and the second largest (or more thoroughly listed) after Numerne with about 3000 individuals. In some locations (NR Motrines ezers, NR Posolnica, NP Laukezers), the dense growth of fir trees has shaded the species, nearly driving it to extinction. Furthermore, a dense moss layer in these and many other locations (e.g., Gauja NP, Kemeri NP, NR Certoka ezers) has contributed to the decline in population. Thus, *P patens* has become extinct in 10 out of the 33 surveyed Natura 2000 areas, while only a few mainly vegetative individuals remained in three others.

Outside Natura 2000 territories, a total of 11,901 individuals were recorded across 44 locations and 277 sites. In 112 sites, where *P. patens* was previously detected, the species was no longer found. The largest population with 4087 *P. patens* was found in Gaigalava MR (Table S1), with *P. patens* growing both in the mature forest and in the new forest along the road edges. The second largest population, Mednu Rubeni (1134 individuals), is also located on the roadside. The study revealed that the highest density of *P. patens* was on roadsides, where the moss layer was not as thick as it was a little further into the forest. In general, the statistical analysis of the data confirmed significant differences between the parameters characterizing the population of *P. patens* on roadsides and in forests. Thus, the 100 m^2^ roadside plots had significantly higher (*t*-test, *p* < 0.05) mean total number of individuals (88.7 vs. 41.4), number of flowering individuals (27.7 vs. 10.5), and number of flowers (63.8 vs. 19.4) compared to forests. Other significant roadside populations of *P. patens* were also found in areas such as Malta–Aglona roadside (719 plants), Pope Micro Reserve (710 plants), outside NP Numerne valnis (427 plants), and others. The largest populations in the forest bordering the highway and railway were surveyed in Darzini (816 plants).

Populations of Ancupani (909 plants), Vezezers (130 plants) and Andrupene (51 plants) grew in light forests on slopes. The small populations (48 and 25 individuals, respectively) of Avotinkalns (on the edge of a steep bank of Daugava river) and Kentes kalns (on the edge of a quarried gravel hill) were threatened by erosion.

A survey of 69 historical sites of *P. patens,* including 26 within Natura 2000, revealed only 10 plants across six historical sites, with the species absent in 63 sites (Figure 1B). Three new sites were discovered in Darzini (Riga) and Kente hill, approximately 30 and 130 m from the historical locations, respectively, which might be remnants of historical populations.

### 3.2. Demographic Assessment of P. patens in Permanent Plots

The four permanent study sites varied in population size and plant concentration. At Darzini, two 10 × 10 m plots located a few 100 m apart contained a total of 268 *P. patens* (2021 data). In Ogres Zilie kalni NP (OZK), 11 plots contained 822 plants, located throughout the nature park. In Kemeri NP (KNP), seven plots contained 93 *P. patens,* more spatially separated from each other, while in Gauja NP (GNP), seven plots contained 38 *P. patens* individuals, located in a wide area from Silciems to Inciems, Lielstraupe and Cesis (Figure 1). Although the sample plots differed and the demographic indicators changed over the years within each plot, the study sites were well characterized by the average number of individuals per 100 m² sample plot: Darzini—134; OZK—75.6; GNP—5.6, KNP—14.1. The number of individuals in GNP and KNP were significantly lower than in the other two, but the differences between Darzini and OZK were not statistically significant (Table 2). 

The average proportion of generative individuals varied significantly in the study sites: 15.0% of individuals flowered in OZK (five-year data), 47.4% in Darzini (five-year data), 37.9% in GNP (four-year data) and 18.1% in KNP (two-year data) (Table 2).

**Figure 2:**
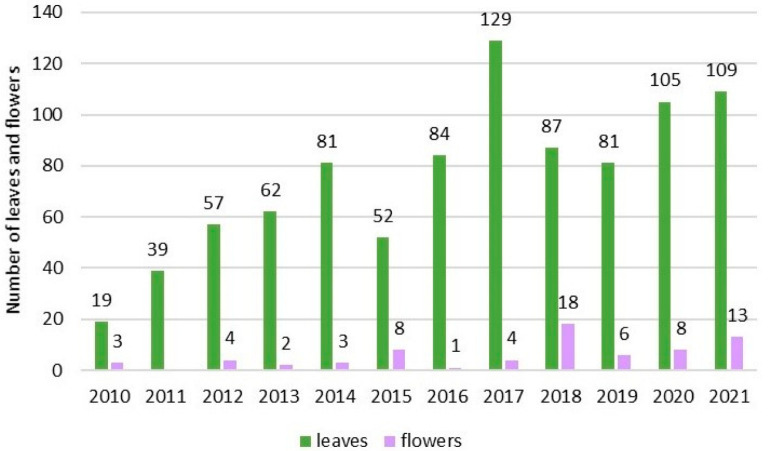
Dynamics of the number of leaves and flowers of one of the largest individuals of *P. patens* in the Darzini sample plot D1 from 2010 to 2021.

Demographic indicators fluctuated significantly over the years, as demonstrated by monitoring of a marked plant in the Darzini sample plot (Figure 2). In addition, a statistically significant trend was found (*r* = 0.882, R^2^ = 0.778, *p* < 0.05) that higher vegetative growth stimulated more flowering in the subsequent year (e.g., 2017/2018).

**Figure 3:**
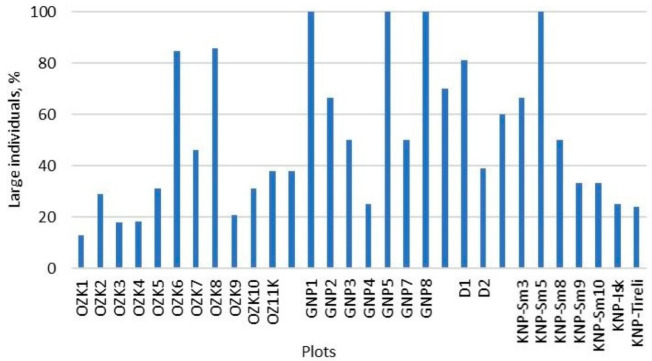
Proportion of large individuals of *P. patens* with ≥10 leaves at four permanent study sites. OZK—Ogres Zilie Kalni Nature Park; GNP—Gauja National Park; KNP—Kemeri National Park, D—Darzini (data from 2017 to 2021).

Across the surveyed populations, demographic indicators and plant age structure varied significantly. OZK was dominated by young plants with 5–10 leaves (Figure 3, Table 2), including nonflowering (juveniles) or individuals with a single flower (young generative individuals). Only two plots (OZK6 and OZK8) had mostly large, mature generative plants averaging two flowers and 19 leaves per individual, and six flowers and 32 leaves per individual, respectively. Large differences in seed viability rates were found between years. Seed viability tests of fully matured OZK seeds showed excellent results in 2018 with viability between 73.5 and 97.3%, but lower rates in 2019 of between 29.1 and 66.9 and in 2021 around 34.2%.

At Darzini, the two sample plots showed contrasting characteristics. Plot D1 was dominated by large, profusely flowering mature generative plants with high morphological productivity but low seed viability of 5.8%, although the average number of flowers in this plot was the highest among the permanent plots (average 442 flowers; five year data). On the other hand, plot D2 had a higher proportion of new generative individuals, with 39% large clumps (2021), 41% of plants that had never flowered in 12 years (mainly with few leaves) and 21% of plants with fewer than 10 leaves that had flowered. This indicates a younger population with a considerable proportion of new generative individuals with high seed viability of 52.7%.

In GNP *P. patens* individuals were spatially distant from each other, with large individuals (≥10 leaves) dominating in 61% of cases. In two plots there was only one large clump (22 and 51 leaves, respectively) and flowering was not abundant (3 and 5 flowers, respectively), suggesting isolated remnants from larger populations and/or a very limited genetic potential. Only in GNP plot 4, young generative individuals dominated with a small number of leaves, 75% of plants had flowered and only 25% had large clumps. GNP4 together with GNP2 (dominated by generative individuals but with higher number of leaves and flowers than GNP4) could be the most promising GNP populations.

In KNP, most plots were characterized by large individuals. However, in the Tireli plot, 40% of individuals were young, nonflowering ones with 2–5 leaves, while only 27% of plants were large clumps with ≥10 leaves. Of these larger individuals, 63% had not flowered in three years, possibly due to unfavorable conditions for flowering, senescence or insufficient observation period.

Since the viability of *P. patens* seeds was analyzed only from plots where the seeds were fully mature, the results are presented for seven study sites (Table 3). Overall, there was little variation in the total number of *P. patens* seeds per inflorescence among these study sites. However, more significant differences were found in seed viability indicators. The highest mean percentage of viable seeds was recorded in OZK, GNP, Mednu Rubeni and Darzini (ranging from 52.7% to 62.4%). Even in study sites where the viability rate was lower, at least one-fifth of the seeds were viable.

### 3.3. Soil Morphology and Chemical Composition

According to soil morphology studies, the soil types in *P. patens* study sites did not differ significantly in typology, as only three soil types and four subtypes were identified: subtypes of typical podzol and illuvial humus podzol from the podzols type, a subtype of sod-podzolic from the podzolic soils type and buried illuvial humus podzol from the anthrosols soil type. The granulometric composition of the soil was determined as sand (fine, medium and coarse sand) in all sample plots.

Characterizing permanent plots in more detail, OZK was dominated by sod-podzolic soil, with only two plots classified as typical podzol. In contrast, the situation in GNP was the opposite, as the typical podzol subtype was predominant (Table S2). The presence of an Ah horizon (mineral horizon below the O horizon) classifies the soil as a sod-podzolic. In KNP, soil studies revealed that typical podzol from the podzol type prevailed, with one exception—Tireli—where a sod-podzolic subtype belonging to the podzolic soil type was identified due to the presence of an Ah horizon (6 cm thick). The average thickness of the accumulation horizon (Ah horizon) in the KNP was much thinner than in OZK and GNP by 90% and 50%, respectively.

The soil subtype in Darzini sample plot next to the railway D2 was classified as illuvial humus podzol due to its low-pronounced upper O horizon (2 cm thick), and a well-pronounced Fe accumulation horizon (Bs). This sample plot exhibited a very high concentration of Fe in the O horizon (2950 mg L^−1^), compared to the average concentration of Fe (627 mg L^−1^) in *P. patens* study sites in Latvia.

Although the differences in O horizon thickness were not large, statistical analysis revealed that these differences were significant (*p* < 0.01) among the four permanent study sites (Table S2).

In general, a wide range of plant-available nutrient concentrations and organic matter content were found across the *P. patens* study sites in Latvia (Table S2). Of the macronutrients, the highest coefficients of variation were recorded for Ca and Mg, while S exhibited the lowest variation. It is interesting that despite the heterogeneity in Ca and Mg content, the smallest differences between sites were found in soil reaction (pH) indicators, thus confirming that most sites were represented by acidic soil: the mean soil pH range for *P. patens* sites in Latvia was 4.07 ± 0.11. Electrical conductivity (EC), which characterizes the concentration of water-soluble ions in the soil extract, was low with average values not exceeding 0.35 ± 0.04 mS cm^−1^ (KNP), indicating generally low levels of readily available macronutrients for *P. patens* populations in Latvia.

Of the micronutrients, Fe and Mn exhibited the highest coefficient of variation (Table S2). Regarding Mn, this likely reflects significant differences in soil organic matter content across *P. patens* sites in Latvia. A statistically significant negative correlation was found between soil organic matter and Mn content (Table S3).

Despite relatively wide ranges of nutrient in the soil across the permanent *P. patens* plots, significant differences between the sites were identified. Higher mean Ca, Mg concentrations and pH levels were found in GNP and Darzini. In the study site Darzini, soils also contained higher concentrations of N, Fe, Mn and Cu. On the other hand, KNP was found to have the lowest P, Fe and B content in soils, as well as the lowest pH level. Nutrient concentrations at OZK and GNP were generally similar.

Overall, pH, EC, organic matter content, and nutrient concentrations (N, P, K, Ca, Mg, S, Fe, Mn, Mo and B) in the soils of the permanent plots (OZK, GNP, KNP, Darzini) fell within the ranges found in other study sites across Latvia. However, the maximum concentrations of Zn and Cu found in soils in OZK, GNP and Darzini were notably higher. Outside these four study sites, *P. patens* soils were characterized by lower mean N and higher P supply.

An important factor influencing plant mineral nutrition is the concentration ratio of nutrients, especially Ca:Mg ratio. The Ca:Mg ratio in soils of the investigated sites ranged from 3.0 to 9.6, in average 5.9 ± 0.2, indicating no major differences in soil parent materials.

To identify associations among soil chemical indices, correlations were calculated between nutrient concentrations, soil pH, EC and organic matter content. In total, 35 out of 120 calculated correlation coefficients (35%) were statistically significant (*p* < 0.05), and only four of them showed negative correlations (Table S3). All statistically significant negative correlations involved soil organic matter content and its correlation with P, Mn, B and pH levels.

Pearson’s correlation coefficients were calculated to assess relationships between soil parameters and the total number of *P. patens* individuals, as well as the number of flowering individuals per 100 m^2^. A significant positive correlation (0.295 ≤ r ≤ 0.413; *p* < 0.05) was found between the number of individuals at a site and soil K, Ca, Mg, B and pH_KCl_ levels (Table S3). In addition, P, Ca, Mn and pH_KCl_ were positively correlated with the number of flowering individuals.

The model variables (K, B, and pH) for the multivariate linear regression analysis were selected based on the significant correlations found with the number of *P. patens* individuals (Table S3). To avoid multicollinearity, Ca and Mg were not included in the model, given the strong correlations observed between pH, Ca, and Mg. Overall, the linear regression model was statistically significant (*p* < 0.05). The three predictors (K, B, pH) significantly contributed to explaining the variability in the number of individuals per site (*p* = 0.002 for K; *p* = 0.016 for B, *p* = 0.001 for pH). The model explained about 35.8% of the variance in number of individuals (R-squared), which suggests a moderate fit. However, the residual standard error of 79.29 indicated that there was still considerable unexplained variability.

Principal component analysis (PCA) was performed to visualize the multivariate space of soil properties and nutrient concentrations for the most studied *P. patens* locations—OZK, GNP, KNP, Darzini, as well as two groups of sites with differing densities of *P. patens* individuals per area (low and high) (Table S4) to assess the potential relationships. PCA results revealed three principal components (eigenvalue > 1.0) that explained 72% of the total variance (Table 4). The most important factors in the PCA of soil chemical composition were soil reaction (pH), electrical conductivity (EC), organic matter content and nutrient levels (Ca, Mg, P, B). Results indicate that PC1 had negative values for almost all variables. The strongest correlation was with soil pH, Ca and Mg levels, suggesting that PC1 reflects soil acidity. PC2 correlates with the compositional elements of soil as total concentration of water-soluble salts (EC) and organic matter content. PC3 had the strongest correlations with nutrients P and B.

**Figure 4:**
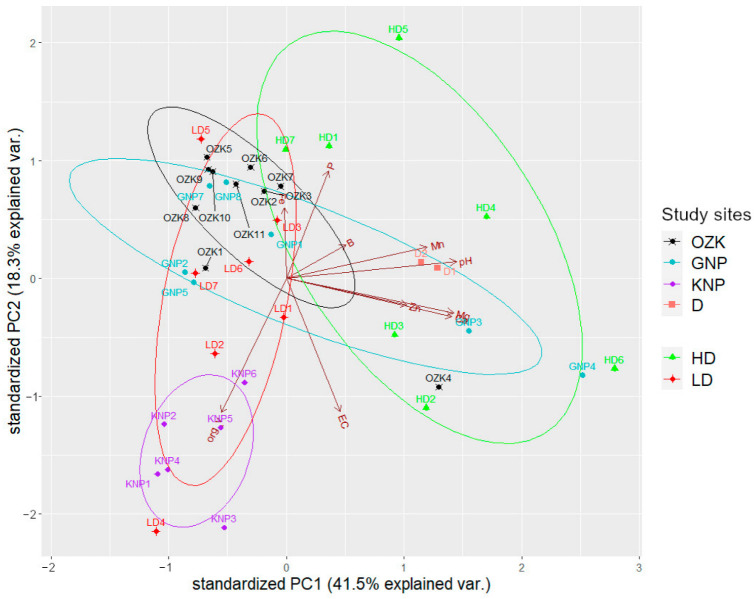
Principal component analysis (PCA) of soil parameters (P, Ca, Mg, Fe, Mn, Zn, Cu, B, pH, EC, organic matter content) describing the soils for the most studied *P. patens* locations—OZK, GNP, KNP, Darzini—as well as two groups of sites with different density of *P. patens* individuals per area (low and high). Low density (LD)—less than 10 individuals per 100 m^2^—and high density (HD)—more than 100 individuals per 100 m^2^. Ellipses show the 95% confidence intervals characterizing groups. OZK—Ogre Zilie Kalni Nature Park; GNP—Gauja National Park, KNP—Kemeri National Park, D—Darzini.

Low and high density populations differed significantly in soil physicochemical properties (Figure 4). Sites with larger population were associated with higher pH and nutrient concentrations, but lower organic matter content. Conversely, sites with smaller populations were associated with lower pH and nutrient levels but higher organic matter content. Among the widely studied sites, KNP was distinct, with the highest organic matter content, the lowest pH, and a small number of individuals per 100 m^2^. Research site Darzini fitted well into the ordination space characterized by a dense population.

Because individual sites of OZK and GNP were scattered in the ordination space, the PCA results confirmed that these study sites did not differ significantly when grouped by soil physicochemical parameters. Although the number of individuals differed significantly among these study sites, 74.5 ± 18.5 and 5.4 ± 2.2, respectively, population size showed little correlation with soil parameters, suggesting the greater importance of other factors.

## 4. Discussion

Carrying out large-scale field work across almost the entire territory of Latvia and conducting numerous laboratory studies, we investigated relationships between *P. patens* population size, demographic indicators, seed viability, and soil parameters.

As a continental climate species, most populations of *P. patens* can be found in the eastern and central part of Latvia (Figure 1). In Western Latvia, where the proximity of the Baltic Sea has a greater influence, *P. patens* is relatively less common. The species was not detected in 10 areas. In total, fifteen small populations with only a few (1 to 8) individuals, twenty-four medium populations (11 to 94 individuals), twenty-five large (>100) and three very large (>1000 individuals) populations have been identified in Latvia (Table S1). When surveying historical sites, we found that many had undergone major habitat changes potentially due to low habitat quality, forestry activities, or inaccuracies in historical data.

The largest number of individuals of the species, which also exhibited higher seed quality, were found near highways, along forest roads and trails, in light forests on slopes, that is, in bright areas. This is consistent with findings from other studies that highlight the importance of good lighting conditions for *P. patens* populations. Under such conditions, populations were larger, with a greater number of flowers and fruits, as well as more flowers per clump [23]. The flowers of *P. patens* exhibit thermonastic and helionastic behavior, with their structure and surface characteristics reflecting sunlight into the flower’s interior, which in sunny weather can increase the temperature inside the flower by 8.9–10.0 °C compared to the surrounding environment, promoting seed production [31,32]. In forests, fewer individuals of *P. patens* were observed, where the plants often had fewer flowers and were overgrown with moss. The natural succession of forests, characterized by nutrient enrichment and increased shading, creates conditions unsuitable for *P. patens,* a trend evident at such study sites as NR Motrines ezers, NR Posolnica, NR Sedas marsh, PLA Ziemelgauja. However, under favorable lighting conditions, the plants flowered, grew into larger individuals and successfully produced new seedlings.

**Figure 5:**
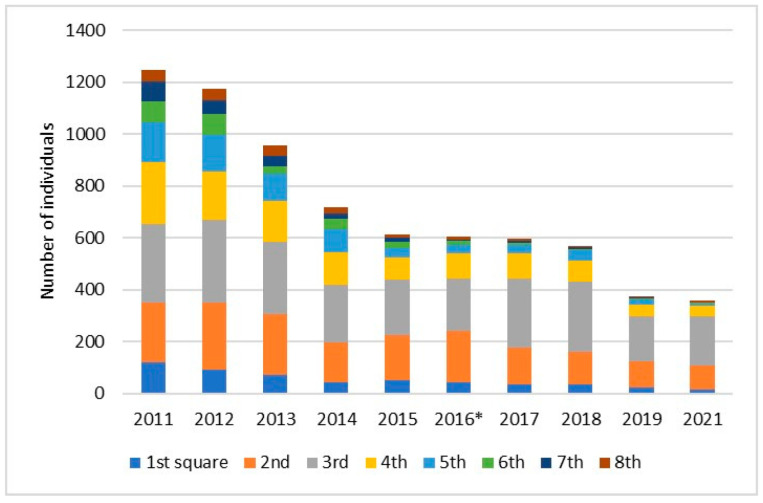
Changes in the number of *P. patens* at the study site Darzini during 10 years in eight 5 m wide squares [20]. * Understory vegetation was removed.

The rapid decline in the number of *P. patens* over the last decade was effectively illustrated by long-term monitoring of a 40 × 40 m sample plot in the Darzini forest (divided into eight 5 × 40 m squares). Light conditions (measured in spring and summer) were on average two times better in squares 2 to 4 than in squares 5 to 8 (with increased understory). In 2016, understory vegetation was removed in Darzini. As a result, a temporary stabilization of the number of *P. patens* individuals was found in the subsequent years (especially in the third square, Figure 5). However, this intervention did not affect the moss layer, and the quick recovery of understory vegetation.

Studies indicated that demographic parameters were negatively correlated with increasing tree cover, especially with the coniferous canopy of boreal forest habitats. Moreover, the dense cover of the moss *Hylocomium splendens* had a more pronounced negative impact on the occurrence of *P. patens* and the demographic parameters of the species than other moss species [33]. The viability of *P. patens* populations is clearly threatened by an increase in ground cover in the absence of natural or anthropogenic disturbance [9].

The demographic status of *P. patens* is closely related to the conditions of each growing site, varies from year to year, and also depends on the creation or consumption of resources in previous years (for example, Figure 2). Demographic parameters can also indicate the status of the population, for example, the D1 population with high morphological quantity (leaves and flowers) and low seed quality indicates that the link with the important function of the plant’s existence, reproduction, has been broken. According to Kricsfalusy, this indicates the aging of the population [21]. A small number of individuals can cause genetic erosion and depression, which negatively impacts quality indicators, such as flowering, seed production and seedling survival [34,35]. Genetic analyses of *P. patens* populations showed that plant genetic diversity is low as populations decline and there is a high level of inbreeding. These factors significantly reduce plant health and viability in small populations [36].

The survival and regeneration of *P. patens* depends on seed production at any given place and time [7], and only viable seeds can provide the basis for new generations and ensure the population’s persistence. The study found that the proportion of viable *P. patens* seeds ranged from 22% to 62% across different geographical areas in Latvia (Table 3) and this level of viability is generally sufficient for the species’ continuation as long as there is also a sufficient number of flowering plants. However, obstacles such as limited open areas and a thick moss layer hinder the sprouting process. It should be noted that sowing in situ is not considered an effective method for strengthening *P. patens* populations due to the very low emergence and survival rates of seedlings [37].

Our study revealed that *P. patens* in Latvia mainly grows in sandy soils, classified as podzols and podzolic soil types. In Estonia, a much greater diversity of soil morphology was characteristic of *P. patens* populations, where, in addition to podzol, brown forest soil, rendzina and coastal gravel was also found [7]. According to Podgorska and Lazarskis [14], the prevailing soil type for *P. patens* in Southern Poland was rendzina, a shallow alkaline or neutral soil. In Canada, populations were found in urban soils, calcareous and eluviated dark brown soils, regosolic, chernozemic and podzolic soils [16]. Thus, based on the wide range of soil types recorded, *P. patens* appears to have a high tolerance to soil conditions.

Our study revealed that soil parameters that most strongly correlated with *P. patens* functional traits were soil pH level and plant available content of nutrients—P, K, Ca, Mg, Mn and B. Although it is reported that a relatively wide range of soil pH is suitable for *P. patens* [7,14,15,16], in Latvia this species typically grows in acidic soils with an average pH of 4.07 ± 0.11. The fact that the average soil pH differed significantly between high and low-density populations suggests that an overly acidic growth environment with correspondingly lower Ca and Mg supply is not favorable for *P. patens*.

In general, *P. patens* in Latvia grows in soils with medium to high organic matter content, although too high content of organic matter seems to be not beneficial. The negative correlation between the organic matter content and soil parameters, which positively affect population status, partially confirms this finding. However, we cannot yet definitely claim a direct relationship between soil organic matter content and population size, as the correlation between soil organic matter content, the number of individuals and flowering individuals was not significantly negative when analyzing all study sites. It would be very valuable to continue and expand the research within the framework of a future project. It should be noted that the organic matter levels obtained in this study fall within the ranges reported by Pilt and Kukk [7] (2002) and Esparrago Llorca [16] for *P. patens* in Estonia and Canada, but were on average twice as high as those reported for *P. patens* in Yellowstone National Park [15].

Although wild plant species are generally considered capable of adapting to a wide range of soil nutrient concentrations [38], sufficient levels of macronutrients in the soil undeniably benefit plant growth, development, and resistance to biotic and abiotic stress. Overall, the low EC of the soil and the absence of elevated salinity indicate a potential nutrient deficiency for *P. patens*. We found that soil fertility indicators such as plant available K and P content were significantly positively correlated with the number of *P. patens* individuals and the number of flowering plants per area.

Because the physiological-metabolic role of K at the plant level is related to the internal transport of substances and energy, with the ability to respond to biotic and abiotic stress, with control of plant growth and metabolism [39], K supply can significantly affect population size and viability. The results of our study regarding the soils of *P. patens* in Latvia also suggest this.

Phosphorous, on the other hand, is essential for promoting plant reproductive growth, including flower and seed formation, while Mg contributes to flower induction process [40]. This could explain the beneficial effects of higher soil P and Mg on both the number of flowering individuals of *P. patens* (Table S2) and the total number of flowers in the plot (r = 0.495 for P, r = 0.253 for Mg; *p* < 0.05).

In addition to macronutrients, the availability of micronutrients in the soil is crucial for the growth, flowering and seed production for endangered species [38]. Our study suggests that for *P. patens* in Latvia, higher soil B content could positively affect the population size, while higher Mn content could promote flowering. For instance, the highest Mn content in the soil (320 mg L^−1^) was recorded in Numerne, where we recorded largest and flower-rich *P. patens* population. Similarly, high Mn concentration in the soil (185 mg L^−1^) was also characteristic of the richest flower sample plot in Gaigalava (636 flowers per 171 individuals), where the second largest population in Latvia was found. The functional basis of this effect could be related to the fact that these micronutrients play a vital role not only in maintaining the overall health and vitality of plants, but also in having a profound effect on the reproductive phases of plants [41].

We would like to end on a hopeful note. This extensive study began in response to a significant decrease in *P. patens* populations in GNP. In 2022, the species was reintroduced to two GNP sites, each with 25 plants grown from seeds obtained from GNP 2 and 4 plots. Observations over three seasons confirmed the success of the reintroduction effort, as some plants flowered.

## 5. Conclusions

Although *P. patens* was not detected in more than a third of the surveyed 624 locations, which covered almost the entire territory of Latvia and in almost none of the historical places, more than 18 thousand individuals of this protected species were counted in total. The fact that the highest number of individuals, in addition to better seed quality, was found near highways, along forest roads and trails, indicated that the growth conditions for *P. patens* were best in places with less competition with other species, better light conditions, and some level of disturbances. In forests, the distribution of *P. patens* was smaller and more scattered, mainly due to increased understory cover and overgrowth by mosses. Significant demographic diversity was found, reflecting in the varying proportions of generative individuals and large clumps, indicating plants at different stages of ontogenesis and the impact of local ecological factors. The typical seed viability of *P. patens* in Latvia, ranging between 22% and 62%, can be considered potentially sufficient for the continuation of the species, as long as there are also enough flowering plants and moss-free space for germination. Although *P. patens* can tolerate a wide soil pH range, in Latvia this species predominantly grows in acidic sandy soils with an average pH_KCl_ of 4.07 ± 0.11. Our study revealed that the soil parameters most strongly positively correlated with *P. patens* local population size and performance included higher soil pH level and greater plant-available nutrient content—P, K, Ca, Mg, Mn and B. It should be noted that an important factor that could have a positive effect on flowering was higher soil P and Mn content. The results also suggest that the soil characteristic most closely linked to low population size of *P. patens* in Latvia could be the high content of soil organic matter.

Finally, through this work, we provide an insight into the current status of *P. patens* in Latvia, the peculiarities of its growing environment and the relationship between these factors, thus contributing to a better understanding of the conditions for the management and/or reintroduction of this endangered plant species. Since the current size of the regional population is still relatively large, the risk of a significant threat to *P. patens* in Latvia in the near future is low. Nevertheless, forest management measures, such as creating open soil areas, where vegetation is removed and shading is reduced, are undeniably necessary to prevent the long-term decline of this species.

## Figures and Tables

**Table 1:** Permanent plots with *Pulsatilla patens* (100 m^2^) for demographic data collection twice a season.

Name of Permanent Sampling Site, Territory	Year of Establishment	No or Name of Plots	Total No of Plots in Site	Sample Collection Period
Rīga, Darzini (D)	2015	1, 2	2	2015–2021
Ogres Zilie kalni Nature Park (OZK)	2017	1–11	11	2017–2021
Gauja National Park (GNP)	2017	1–5	7	2017–2021
	2018	7, 8		2018–2021
Kemeri National Park (KNP)	2019	Iskopi, Tireli, Smarde 3	7	2019–2021
	2020	Smarde 5, 8, 9,10		2020–2021

**Table 2:** Demographics of *Pulsatilla patens* at the permanent study sites (per 100 m² plot) in Latvia, 2017–2021.

Location	Number of Samples ^1^	Total Number of Individuals	Number of Flowering Individuals	Flowering Individuals %	Total Number of Flowers in the Sample Plot	Number of Flowers per Plant	Number of Leaves per Plant
OZK	55	75.6 ± 8.1 c ^2^	11.3 ± 1.1 b	15.0	16.1 ± 1.5 b	1.6 ± 0.2 a	11.8 ± 1.0 a
GNP	28	5.6 ± 0.9 a	2.4 ± 0.5 a	37.9	5.5 ± 1.2 a	2.4 ± 0.3 ab	18.0 ± 2.4 b
KNP	21	14.4 ± 5.6 b	2.7 ± 0.7 a	18.11	3.8 ± 0.9 a	1.4 ± 0.2 a	12.5 ± 1.4 a
Darzini	14	133.9 ± 44.9 c	63.6 ± 15.1 c	47.4	218.6 ± 66.5 c	2.8 ± 0.3 b	18.9 ± 2.8 b

^1^ The number of plots per site multiplied by the number of sampling times, according to Table 1. ^2^ Means with different letters in the column were significantly different (*t*-Test, *p* < 0.05). OZK—Ogres Zilie Kalni Nature Park; GNP—Gauja National Park; KNP—Kemeri National Park.

**Table 3:** Total number of seeds per infructescence and percentage of viable seeds of *P. patens* in the study sites in Latvia, 2018–2021.

Study Site	Number of Infructescences	Number of Seeds per Infructescence	Viable Seeds (%) ^1^
Ancupani	10	147 ± 8.8 ab ^2^	23.2 ± 2.9 a
Ape	10	139.1 ± 13.4 ab	42.07 ± 6.1 b
Darzini	42	136.2 ± 15.5 ab	52.7 ± 6.5 b
Driksnas sils	4	123 ± 28.7 a	21.85 ± 5.9 a
Gaigalava	15	108.3 ± 11.2 a	33.8 ± 5.2 ab
GNP	14	71.5 ± 16.5 a	60.9 ± 10.9 b
Mednu Rubeni	14	130.0 ± 28.2 ab	59.2 ± 13.2 b
OZK	37	99.7 ± 6.7 a	62.4 ± 3.2 bc

^1^ Only mature seeds analyzed. OZK—Ogres Zilie Kalni Nature Park, GNP—Gauja National Park. ^2^ Mean values with different letters in the column were significantly different (*t*-Test, *p* < 0.05).

**Table 4:** Eigenvector values of principal component analysis (PCA) of soil samples describing correlations with the first three components.

Variable	PC1	PC2	PC3
P	−0.116	−0.446	0.583
Ca	−0.457	0.158	0.016
Mg	−0.461	0.142	0.034
Fe	0.006	−0.293	0.020
Mn	−0.388	−0.130	0.172
Zn	−0.333	0.109	−0.295
pH	−0.469	−0.070	0.090
EC	−0.148	0.553	0.247
B	−0.165	−0.140	−0.666
Organic matter	0.179	0.555	0.160
Explained variance	41.5%	18.3%	12.4%

## Data Availability

All data reported here are available from the authors upon request.

## Supplementary Materials

The following supporting information can be downloaded at: https://www.mdpi.com/article/10.3390/plants14030375/s1, Table S1: Number of individuals of *Pulsatilla patens* and surveyed sites in and outside Natura 2000 territories in Latvia; Table S2: Nutrient concentration (mg L^−1^, 1M HCl extraction), soil pHKCl, electrical conductivity (EC, mS cm^−1^), and organic matter content in air-dried soil samples (upper topsoil horizon), thickness of soil horizons, as well as number of individuals per 100 m^2^ from *P. patens* study sites in Latvia, 2020–2021; Table S3: Pearson’s correlation matrix between soil chemical datasheet (nutrient concentrations, soil pH, electrical conductivity, organic matter content) and number of individuals and flowering individuals per 100 m^–2^ from *P. patens* study sites in Latvia; Table S4: Nutrient concentration (mg L^−1^, 1M HCl extraction), soil pHKCl, electrical conductivity (EC, mS cm^−1^), and organic matter content in air-dried soil samples (upper topsoil horizon), as well as number of individuals per 100 m^2^ from high and low density *P. patens* study sites in Latvia, 2020–2021.
