# Supplementary material for: Current Status of Pulsatilla patens in Latvia—Population Size, Demographic and Seed Viability Indicators, Soil Parameters and Their Relationships
Source: Plants (Basel). 2025 Jan 26;14(3):375. doi: 10.3390/plants14030375 (PMC11819818; doi:10.3390/plants14030375)
Supplement: Supplementary file 1 [file plants-14-00375-s001.zip › supplementary_data_revision_final.pdf]

Table S1. The number of individuals of *Pulsatilla patens* (*P.p.*) and surveyed sites in and outside Natura 2000 territories in Latvia.

No.	Natura 2000 territories	Total surveyed *P.p.* sites/ sites without *P.p.*	Number of sample plots	Total number of individuals	No.	Outside Natura 2000 territories	Total surveyed *P.p.* sites/ sites without *P.p.*	Number of sample plots	Total number of individuals
1	NR Krustkalni	3	1	149	1	Pope Zagarkalns	4	1	105
2	NR Teici	6	1	649*	2	Popes Micro reserve	7	1	710
3	Gauja National park	8	7	43	3	Turn to Valdemarpils	2		140
4	Kemeri National park	12	7	160	4	Vezezers	3	1	130
5	NP Daugavas ieleja	2/2		0	5	Riga Bikernieki, Smerlis, Bergi	19/9	2	42
6	NP Silene	18/7	1	70	6	Riga Darzini	35/16	2	816
7	NP Tervete	1		2	7	Planupe	1	1	25
8	NP Istras pauguraine			0	8	Smiltene-Silva roadside	2	1	19
9	NP Dolessala	1		0	9	Strenci-Valka roadside	2	1	115
10	NP Engures ezers			0	10	Madona-Iedzeni roadside	1		120
11	NP Numernes valnis	8	3	3000*	11	Oleri Micro reserve	9/6		6
12	Razna NP	2	1	555*	12	Turki Micro reserve	3	1	252
13	NP Driksnas sils	8	2	107*	13	Outside Turki Micro reserve	1		178
14	NP Laukezers	7/1	1	14	14	Turki roadside	3		71
15	NP Ogres Zilie kalni	25/6	11	854	15	Gaini roadside	1		23
16	NR Certoka ezers	15/3	1	36	16	Steki roadside	1	1	25
17	NR Greblukalns	11/5		16	17	Steki young forest	2		30
18	NR Lielie Kangari	6/4		8	18	Gaigalava Micro reserve	5	3	4087
19	NR Plienciema kapa	1/1		0	19	Skrebeļi, Rozupe parish	1		11
20	NR Vecdaugava	1/1		0	20	Idena	1	1	22
21	NR Garkalnes mezi	4/4		0	21	Outside NP Numernes valnis	1		427
22	NR Klintaine	4/2		94	22	Ancupani	6/2	1	909
23	NR Motrines ezers	4/3		1	23	Andrupene	1	1	51
24	NR Posolnica	68/56	1	21	24	Malta-Aglona roadside	2		719
25	PLA Augsdaugava	65/27	2	496	25	Daugmale	4	1	56
26	PLA Ziemelgauja	5/5		0	26	Taurkalne	7/6		115
27	PLA Adazi	21/8		202	27	Vecumnieki	6/5		13
28	NP Piejura	3/3		0	28	Mednu Rubeni	5	1	1134
29	NP Salacas ieleja	10/0		29	29	Ape	6		68
30	NP Ogres ieleja	1/1		0	30	Avotinkalns	3		48
31	NR Sedas purvs	2/1		2	31	Garkalne	6/4		112
32	NR Timsmales ezers	13/8		13	32	Ķente hill	2/1		25
33	NR Lubana mitrajs	5/1	1	107*	33	Outside NR Certoka ezers	18/1		68
					34	Outside NR Motrines ezers	34/17		61
					35	Outside NR Posalnica	48/32		39

36	Daugavpils Liepziedi	2	483
37	Daugavpils Rugeli	3	575
38	Daugavpils Skirotava	2	58
39	Lube	4/1	6

* The number of *P. patens* individuals also includes data obtained by other Latvian experts in additional sites nearby. NR – Nature reserve; NP – Nature park; PLA – Protected landscape area

Table S2. Nutrient concentration (mg L^{-1}, 1M HCl extraction), soil pHKCl, electrical conductivity (EC, mS cm^{-1}), and organic matter content in air-dried soil samples (upper topsoil horizon), thickness of soil horizons, as well as and number of individuals per 100 m^2 from *P. patens* study sites in Latvia, 2020−2021.

	**OZK** ***n*=11**		**GNP** ***n*=7**		**KNP** ***n*=7**		**Darzini** ***n*=2**		**Other study sites** **n=33**	
	**Mean ± SE Range**	**CV[1]**	**Mean ± SE Range**	**CV**	**Mean ± SE Range**	**CV**	**Mean ± SE Range**	**CV**	**Mean ± SE Range**	**CV**
**N**	34.2 ± 5.3 b [2]	51.44	34.1 ± 5.5 b	42.89	47.7 ± 5.8 b	29.84	73.0 ± 25.0 bc	48.43	14.7 ± 2.3 a	**89.42**
	8–59		13–50		33–68		48–98		5–65	
**P**	56.8 ± 5.8 b	33.70	62.4 ± 10.4 b	44.27	28.7 ± 2.85 a	24.34	51.5 ± 0.5 b	1.37	76.9 ± 9.7 c	**71.47**
	31−100		33−109		22–41		51–52		15−267	
**K**	49.6 ± 7.4 a	49.65	56.1 ± 2.4 a	11.29	81.7 ± 5.85 b	17.57	53.5 ± 1.5 a	3.97	74.2 ± 7.7 b	58.46
	34−120		44–65		64–100		52–55		39−273	
**Ca**	362.3 ± 93.5 a	**85.61**	1002.0 ± 432.1 b	**114.01**	376.7 ± 49.44 a	32.15	1315.0 ± 120.0 bc	12.91	889.9 ± 112.8 b	**71.68**
	140−1260		140−2950		230–570		1195–1435		326−2503	
**Mg**	76.9 ± 22.2 a	**95.53**	164.7 ± 63.4 ab	**101.80**	74.7 ± 17.4 a	57.17	247.5 ± 27.5 c	15.71	161.0 ± 26.4 b	**92.60**
	35−295		41−450		37−155		220−275		48−570	
**S**	10.18 ± 0.55 a	18.01	9.29 ± 1.04 a	29.63	8.97 ± 1.04 a	28.31	20.00 ± 8.00 a	56.57	9.36 ± 0.53 a	32.22
	7−14		5−13		5.7−12		12−28		4.4−16	
**Fe**	950.9 ± 49.9 b	28.01	723.6 ± 103.95 b	38.01	173.3 ± 54.6 a	77.14	1845.0 ± 1105.0 b	**84.70**	746.6 ± 162.5 b	**123.09**
	290−850		440−1150		70−430		740−2950		119−4749	
**Mn**	49. 0± 10.4 a	**70.42**	70.6 ± 17.2 ab	**64.41**	32.9 ± 10.8 a	**80.43**	135.0 ± 10.0 b	10.48	102.1 ± 14.9 b	**82.76**
	6−100		10.5−130		6.5−76.5		125−145		17.5−320	
**Zn**	5.76 ± 0.94 b	**53.96**	6.41 ± 1.84 ab	**76.09**	4.40 ± 0.10 a	5.79	13.50 ± 0.50 c	5.24	5.8 5± 0.32 b	30.83
	2.8−13		3−15.5		4−4.7		13−14		2.65−11	
**Cu**	1.14 ± 0.13 ab	37.18	1.16 ± 0.28 a	**63.44**	0.77 ± 0.05 a	15.80	6.00 ± 3.50 c	**82.50**	0.8 0± 0.05 a	36.55
	0.6–9		0.5−2.6		0.6−0.95		2.5−9.5		0.4−1.95	
**Mo**	0.0 5 ± 0.01 b	40.69	0.03 ± 0.003 a	23.01	0.03 ± 0.002 a	15.49	0.04 ± 0.00 b	0.00	0.06 ± 0.01 b	75.28
	0.03–0.09		0.02–0.04		0.03–0.04		0.04–0.04		0.02–0.23	

**B**	0.50 ± 0.06 b	40.00	0.19 ± 0.03 a	48.45	0.15 ± 0.03 a	55.78	0.60 ± 0.10 b	23.57	0.42 ± 0.03 b	37.23
	0.1–0.8		0.1–0.3		0.1–0.3		0.5–0.7		0.1–0.7	
**Na**	7.1 ± 0.25 a	11.72	9.9 ± 1.89 a	50.48			13.0 ± 1.0 b	10.88	12.7 ± 0.9 b	40.62
	9–9		6–19.5				12–14		6.4–32.5	
**pH_{KCl}**	3.8 5± 0.12 b	10.14	4.24 ± 0.50 b	30.94	3.31 ± 0.12 a	9.24	4.62 ± 0.32 b	9.80	4.21 ± 0.15 b	20.61
	3.45–4.75		3.24–6.27		2.94–3.78		4.30–4.95		3.20–6.15	
**EC**	0.18 ± 0.02 a	40.70	0.2 3± 0.02 a	19.32	0.35 ± 0.04 b	28.21	0.28 ± 0.03 ab	12.86	0.30 ± 0.02 b	35.56
	0.13–0.39		0.18–0.31		0.28–0.54		0.25–0.30		0.17–0.70	
**Organic**	6.77 ± 0.56 a	27.66	8.69 ± 0.57 b	17.34	39.70 ± 5.12 d	31.61	10.75 ± 3.35 a	44.07	19.17 ± 2.87 c	**84.82**
**matter, %**	5.2–12		7.7–12		21.5–55.13		7.4–14.1		4.1–80	
**O horizon, cm**	3.45 ± 0.3 b<br>2-5	32.66	5.14 ± 0.7 c<br>2-8	36.25	8.43 ± 0.6 d<br>5–10	20.39	2.50 ± 0.5 a<br>2-3	28.28	4.00 ± 0.3 b<br>1-8	46.23
**Ah horizon, cm**	15.91 ± 3.2 c<br>0–29	66.19	3.14 ± 1.8 a<br>0–12	155.26	1.57 ± 0.9 a<br>0-6	146.30	4.00±1.0 ab<br>3-5	35.36	3.00 ± 1.1 a<br>0-31	203.20
**Individuals**	74.5 ± 18.5 b<br>9–163	82.27	5.4 ± 2.2 a<br>1–15	104.67	15.0 ± 11.3 a<br>1–71	184.13	134 ± 44.0 b<br>90–178	46.44	81.9 ± 20.7 b<br>1–555	142.74

[1] Coefficient of variation. [2] Means with different letters in a row were significantly different between study sites (t-test, $p < 0.05$, a < b). (OZK – Ogres Zilie Kalni Nature Park; GNP – Gauja National Park; KNP – Kemeri National Park).

Table S3. Pearson's correlation matrix between soil chemical datasheet (nutrient concentrations, soil pH, electrical conductivity, organic matter content) and number of individuals and flowering individuals per 100 m^2 from *P. patens* study sites in Latvia.

	**N**	**P**	**K**	**Ca**	**Mg**	**S**	**Fe**	**Mn**	**Zn**	**Cu**	**Mo**	**B**	**Na**	**pH_{KCl}**	**EC**	**Org. mat.**	**Individuals**
**P**	-0.086																
**K**	0.022	-0.027															
**Ca**	-0.012	0.067	0.045														
**Mg**	-0.045	0.128	0.012	**0.943***													
**S**	**0.567***	0.065	0.028	0.075	0.044												
**Fe**	0.183	0.124	-0.085	-0.083	-0.125	**0.274***											
**Mn**	-0.092	**0.321***	-0.046	**0.484***	**0.479***	0.122	0.108										
**Zn**	0.277	-0.086	0.027	**0.627***	**0.489***	**0.359***	-0.009	**0.283***									
**Cu**	**0.541***	-0.020	-0.102	**0.248***	0.175	**0.663***	**0.344***	0.108	**0.593***								
**Mo**	-0.166	0.092	-0.132	-0.013	-0.043	0.159	-0.119	**0.464***	0.044	-0.102							
**B**	-0.220	-0.096	-0.052	0.173	0.240	0.001	-0.045	0.190	**0.250***	0.154	0.127						
**Na**	-0.155	-0.141	0.118	**0.375***	**0.278***	**0.255***	-0.178	0.210	**0.359***	0.090	0.230	0.108					
**pH_{KCl}**	-0.077	**0.358***	-0.074	**0.866***	**0.880***	-0.029	-0.079	**0.567***	**0.553***	0.187	0.050	0.240	0.177				

**EC**	0.187	-0.204	**0.328***	0.291*	**0.273***	**0.387***	-0.189	0.075	0.153	-0.040	0.021	-0.144	**0.508***	-0.018			
**Org. mat.**	0.111	**-0.411***	0.245	-0.094	-0.129	0.057	-0.133	**-0.331***	-0.159	-0.135	-0.201	**-0.252***	0.311*	**-0.399***	**0.542***		
**Individuals**	-0.063	0.147	**0.308***	**0.295***	**0.413***	0.047	-0.110	0.218	0.082	0.041	0.079	**0.350***	0.064	**0.412***	0.054	-0.150	
**No of flow. individuals**	0.033	**0.480***	0.034	0.185	**0.281***	0.100	0.000	**0.321***	0.087	0.056	0.086	0.107	0.086	**0.400***	0.007	-0.146	**0.610***

The colour intensity of squares is proportional to Pearson's correlation coefficients, red colour indicates positive correlations and blue—negative correlations. Asterisk (*) indicates significance at $p < 0.05$, $r > 0.250$, $n = 60$.

Table S4. Nutrient concentration (mg L^{-1}, 1M HCl extraction), soil pHKCl, electrical conductivity (EC, mS cm^{-1}), and organic matter content in air-dried soil samples (upper topsoil horizon) as well as and number of individuals per 100 m^2 from high and low density *P. patens* study sites in Latvia, 2020–2021.

	**High density populations (n=7)**		**Low density populations (n=7)**	
	**Mean ± SE**	**CV[1]**	**Mean ± SE**	**CV**
**N**	14.0 ± 4.1 a [2]	77.48	18.6 ± 4.0 a	56.63
**P**	112.7 ± 32.2 a	75.69	75.9 ± 22.0 a	76.63
**K**	93.9 ± 30.7 a	86.43	74.9 ± 11.7 a	41.39
**Ca**	1324.14 ± 258.9 b	51.73	406.9 ± 26.4 a	17.18
**Mg**	305.86 ± 71.4 b	61.80	64.0 ± 6.0 a	24.77
**S**	8.6 ± 0.7 a	20.16	11.3 ± 0.7 a	16.37
**Fe**	584.6 ± 94.2 a	42.64	1296.0 ± 614.6 a	125.46
**Mn**	154.4 ± 32.2 b	55.08	46.6 ± 12.1 a	68.37
**Zn**	5.27 ± 0.57 a	28.76	4.51 ± 0.38 a	22.31
**Cu**	0.78 ± 0.04 a	13.29	0.80 ± 0.12 a	40.02
**B**	0.49 ± 0.06 b	40.18	0.34 ± 0.05 a	37.11
**pH_{KCl}**	5.20 ± 0.30 b	15.52	3.51 ± 0.09 a	6.60
**EC**	0.28 ± 0.04 a	34.34	0.30 ± 0.02 a	17.99
**Organic matter, %**	10.76 ± 2.09 a	51.43	26.75 ± 9.16 b	90.57
**Individuals**	255.7 ± 54.3 b	56.14	4.0 ± 1.0 a	67.70
**No. of flowering individuals**	61.57 ± 19.67 b	84.53	3.57 ± 0.90 a	66.37

[1] Coefficient of variation. [2] Means with different letters in a row were significantly different between high (more than 100 individuals per 100 m^2) and low density (less than 10 individuals per 100 m^2) study sites (t-test, $p < 0.05$, a < b).
